# Characterization of Hemorrhagic Fever with Renal Syndrome Caused by Hantaviruses, Estonia

**DOI:** 10.3201/eid1311.070221

**Published:** 2007-11

**Authors:** Irina Golovljova, Veera Vasilenko, Vassili Mittženkov, Tiina Prükk, Elviira Seppet, Sirkka Vene, Bo Settergren, Alexander Plyusnin, Åke Lundkvist

**Affiliations:** * National Institute for Health Development, Tallinn, Estonia; † Swedish Institute for Infectious Disease Control, Solna, Sweden; ‡ Karolinska Institutet, Stockholm, Sweden; § University of Tartu, Tartu, Estonia; ¶ Central Hospital, Kristianstad, Sweden; # University of Helsinki, Helsinki, Finland

**Keywords:** Hemorrhagic fever with renal syndrome, nephropathia epidemica, hantaviruses, clinical symptoms, epidemiology, dispatch

## Abstract

Thirty cases of hemorrhagic fever with renal syndrome (HFRS) due to Puumala virus (PUUV), Saaremaa virus (SAAV), and Dobrava virus infection were confirmed in Estonia. Except for the levels of serum creatinine, no remarkable differences were found in the clinical course of HFRS caused by PUUV and SAAV.

Hemorrhagic fever with renal syndrome (HFRS) is an endemic zoonosis in Eurasia. In Europe, HFRS is caused by 3 hantaviruses: Puumala virus (PUUV), carried by the bank vole (*Myodes glareolus*); Dobrava virus (DOBV), carried by the yellow-necked mouse (*Apodemus flavicollis*); and Saaremaa virus (SAAV), carried by the striped field mouse (*A. agrarius*) (*1*).

In Estonia, PUUV and SAAV were found in wild rodents. High prevalence of human antibodies has been detected against SAAV (23%) on Saaremaa Island and against PUUV (18%) in central Estonia (*2*,*3*). In contrast to Slovenia, Croatia, and Bosnia-Herzegovina, where a milder HFRS caused by PUUV and a more severe form caused by DOBV have been reported (*4*,*5*), no severe forms of HFRS have been observed in Estonia thus far. Our first report of HFRS in Estonia included cases caused by PUUV and SAAV (*6*). (At that time, SAAV was mistakenly designated DOBV; therefore, DOBV and SAAV infections could not be distinguished.)

No fatal cases have been reported on Saaremaa Island, where 23% of the population has antibodies against SAAV; this finding strongly suggests that SAAV causes a milder form of HFRS. To validate this hypothesis, we analyzed clinical data and laboratory findings in Estonian HFRS patients infected by different hantaviruses.

## The Study

During 1999–2004, serum samples from 321 patients with suspected HFRS were analyzed by using ELISA. Of these, 30 were confirmed for hantavirus infection by the Department of Virology, National Institute for Health Development (Tallinn). Blood samples were collected from patients during the acute (at admission to a hospital) and convalescent phases (Table 1). The acute-phase serum samples were tested by immunoglobulin (Ig) M and IgG ELISA as described (*7*,*8*). Convalescent-phase serum samples were collected from 25 patients, and the causative hantavirus was typed by focus reduction neutralization test (FRNT) as described earlier (*7*). We found that 21 patients were infected by PUUV, 3 by SAAV, and 1 by DOBV (at least a 4-fold higher endpoint titer).

**Table 1:** Hantavirus-specific antibodies in 30 HFRS patients in Estonia as determined by ELISA and FRNT*

Patient no.	ELISA				FRNT of convalescent-phase serum				
	Time after onset, d	PUUV IgM/IgG	SAAV IgM/IgG		Time after onset	PUUV	SAAV	DOBV	SEOV
681	13	**1.095/0.556†**	0.075/0		5 mo	**640‡**	40	<40	<40
715	12	**1.147/0.258**	0.052/0.045		21 d	**640**	<40	<40	<40
716	17	**1.072/0.383**	0.028/0		1 mo	**2,560**	<40	<40	<40
718	7	**1.079/0.142**	0/0.019		1 mo	**2,560**	<40	<40	<40
728	24	**0.602/0.727**	0.039/**0.145**		9 mo	**640**	<40	<40	<40
731	8	**1.43/0.309**	0/**0.102**		24 d	**2,560**	<40	<40	<40
761	9	**1.048/0.213**	0.034/**0.138**		ND	ND	ND	ND	ND
763	9	**1.309/0.631**	0.031/0.088		ND	ND	ND	ND	ND
766	15	**1.340/0.586**	0.036/0.043		7 mo	**640**	<40	<40	<40
767	16	**1.840/0.627**	0.040/0.034		12 mo	**640**	<40	<40	<40
770	13	**0.995/0.127**	**0.231/**0.031		5 mo	**160**	<40	<40	<40
772	6	**0.331/**0.044	**0.137**/0.021		11 mo	**160**	<40	<40	<40
774	4	**1.361/0.863**	**0.103**/0.088		11 mo	**160**	<40	40	<40
800	8	**1.151/0.152**	0/0.099		ND	ND	ND	ND	ND
812	9	**1.144/0.244**	0.026/0.046		9 mo	**640**	<40	<40	<40
813	7	**1.058/**0.089	0.082/0.06		2 mo	**640**	<40	<40	<40
840	8	**0.724**/0.038	0.083/0.055		9 mo	**640**	<40	<40	<40
841	8	**0.704/0.142**	0.073/0.013		20 d	**2,560**	160	160	<40
855	24	**0.791/0.152**	**0.632/**0.090		1 mo	**640**	40	40	<40
857	5	**1.133**/0.043	**1.053**/0.067		ND	ND	ND	ND	ND
861	23	**1.051/0.184**	**0.559**/0.035		1.5 mo	**640**	<40	<40	<40
895	5	**0.957/0.135**	ND/0.011		1 mo	**2,560**	<40	<40	<40
899	13	**0.797/0.120**	ND/0.005		1.5 mo	**160**	<40	<40	<40
927	11	**0.833/0.279**	ND/0.004		1.5 mo	**640**	<40	40	<40
933	5	**0.569/0.464**	ND/**0.116**		18 mo	**160**	<40	40	<40
691	6	**0.355**/0.047	**1.014**/0.081		4 mo	<40	**160**	40	<40
769	10	**0.271**/0	**0.218/0.254**		20 d	<40	**2,560**	640	<40
801	14	0/0.038	**0.394/1.548**		1.5 mo	<40	**640**	160	<40
849	4	0.011/0	**0.599/0.133**		ND	ND	ND	ND	ND
795	12	**0.144**/0	**0.442/362**		11 mo	<40	160	**640**	<40

*HFRS, hemorrhagic fever with renal syndrome; FRNT, focus reduction neutralization test; PUUV, Puumala virus; SAAV, Saaremaa virus; Ig, immunoglobulin; DOBV, Dobrava virus; SEOV, Seoul virus; ND, not done. Numbers in **boldface** indicated positive results in ELISA and at least 4-fold higher titer in FRNT. The cut-off value for ELISA was set at OD = 0.100. †Mean optical density (OD) for duplicate samples. ‡Reciprocal endpoint titer at an 80% reduction.

Complete clinical documentation was available for 25 patients, and their case records were reviewed. One patient with PUUV infection, a 1-year-old child, was excluded from our study.

The most common clinical symptoms were acute onset of disease, fever, and back pain (Table 2). Impaired renal function was also noted in almost all patients. Proteinuria and microscopic hematuria were reported for all PUUV- and SAAV-infected patients, but hematuria was not found in the DOBV-infected patient. Oliguria (<400 mL/24 h) was noted for 55% of the PUUV patients and all SAAV patients; subsequent polyuria (>2,500 mL/24 h) was noted for 45% of the PUUV-infected patients and in 67% of the SAAV patients. Mild oliguria (380 mL/24 h) and polyuria (5,000 mL/24 h) also developed in the patient with DOBV infection. One patient with PUUV infection and 1 with SAAV infection required hemodialysis because of symptoms of uremia. An increased level of serum creatinine was found in all patients with PUUV and SAAV infections. The mean level of serum creatinine was significantly higher in the SAAV patients (p = 0.043).

**Table 2:** Frequency of clinical symptoms and laboratory findings in 24 HFRS patients, Estonia*

Findings	No. patients (%)		
	PUUV, n = 20 (%)	SAAV, n = 3 (%)	DOBV, n = 1
Signs and symptoms			
Fever	20 (100)	3 (100)	1
Headache	8 (40)	3 (100)	0
Abdominal pain	11 (55)	3 (100)	1
Back pain	16 (80)	2 (67)	1
Nausea/vomiting	11 (55)	2 (67)	1
Hemorrhage	2 (10)	1 (33)	0
Visual impairment	5 (25)	0	0
Diarrhea	3 (15)	2 (67)	1
Oliguria (<400 mL/d)	11 (55)	3 (100)	1
Polyuria (>2,500 mL/d)	9 (45)	2 (67)	1
Hematuria (microscopic)	20 (100)	3 (100)	0
Hemodialysis	1 (5)	1 (33)	0
Laboratory findings	Mean value (% patients with abnormal findings), range		
Leukocyte count, 4–10 × 10^9^/L	11 (58), 5.7–24.4	9 (33), 8.1–11.3	5
Thrombocyte count, 100–400 × 10^9^/L	120 (57), 12.6–350	126 (33), 90–163	174
C-reactive protein <10 mg/L	84 (94), 9.7–229.8	69 (100), 52–95	168
Serum urea, 2.8–8.3 mmol/L	23 (94), 5.0–87.0	31 (100), 20.1–42.5	ND
Serum creatinine, 35–115 μmol/L	303 (100), 120–1,124	606 (100)†, 489–796	98
AST <26 U/L	74 (100), 28–205	56 (100), 39–77	122
ALT <35 U/L	62 (88), 20–233	71 (100), 41–123	ND
Proteinuria (g/L)	3 (95), 0.25–11.8	7 (100), 1.06–17.8	1

*HFRS, hemorrhagic fever with renal syndrome; PUUV, Puumala virus; SAAV, Saaremaa virus; DOBV, Dobrava virus; AST, aspartate aminotransferase; ALT, alanine aminotransferase. †Statistically significant result, p<0.05.

Other common abnormalities in laboratory findings were the elevated levels of C-reactive protein, serum urea, and transaminases. We observed normal mean levels of platelets; however, thrombocytopenia was found in 57.1% of the PUUV-infected patients and only in 33% of the SAAV-infected patients.

In summary, no remarkable differences in the clinical course of HFRS caused by PUUV and SAAV were found. The comparison between the 3 groups was difficult because of the small number of patients infected by SAAV and DOBV. The only patient with DOBV infection demonstrated a mild clinical course of disease.

## Conclusions

Because at least 2 hantaviruses, PUUV and SAAV, circulate in Estonia, our main aim was to describe the clinical courses of HFRS caused by different hantaviruses. We found that HFRS cases caused by PUUV, SAAV, and DOBV all occurred in Estonia. Approximately 75% of the Estonian patients were infected with PUUV, a finding that agrees with the more frequent detection of hantavirus antigen in *M. glareolus* in mainland Estonia. DOBV antibodies have been detected in HFRS patients (the present study) and in blood donors (*9*), but the virus has thus far not been detected in rodents captured in Estonia.

Hallmarks of HFRS, such as blurred vision and hemorrhages, were not frequent among the patients in our study. Although acute transient myopia was observed in 25% of PUUV-infected patients, the differences between the groups were not statistically significant. In Fennoscandia, where only PUUV infections have been reported, visual dysfunction has been reported for 31%–36% of the patients (*10*,*11*). In Slovenia, 83% of PUUV-infected patients and 42% of DOBV-infected patients reported blurred vision (*4*). The reason for different prevalence rates of this symptom between countries is unclear, but the type of causative hantavirus or lack of a diagnosis of visual disturbance might play a role. Hemorrhagic manifestations were found in 3 patients (2 PUUV and 1 SAAV), but no statistically significant differences were found between the groups. The patient with a DOBV infection did not show any signs of hemorrhages. Normal levels of mean platelet counts were found in all groups of our HFRS patients, although in 57% and 33% of PUUV- and SAAV-infected patients, respectively, a mild thrombocytopenia was found. Slovenian DOBV-infected patients had significantly lower platelet counts than PUUV-infected patients (*4*). Thrombocytopenia was also observed for 75% and 52% of patients in Finland and Sweden, respectively (*10*,*11*).

Gastrointestinal symptoms are common in hantavirus infections. We did not, however, find significant differences between the PUUV- and SAAV-infected patients, although diarrhea and abdominal pains were more common in SAAV- than PUUV-infected patients. In our study, transient impairment of renal function was noted in all patients, together with elevated levels of serum urea and creatinine, findings in line with those from previous studies in other countries. Our study demonstrated that HFRS symptoms were not generally distinguishable between the PUUV- and SAAV-infected patient groups. Only the level of serum creatinine was significantly higher in the SAAV-infected patients.

Two forms of HFRS have been reported from the Balkans, a mild form with mortality rates up to 0.1% caused by PUUV, and a severe form with mortality rates >10% caused by DOBV. On the other hand, in Estonia and other countries such as Germany, Denmark, Slovakia, Latvia, and Lithuania, where SAAV (or DOBV-Aa, as SAAV is occasionally designated) circulates, no fatal cases have been reported.

The symptoms caused by different hantaviruses range from subclinical or mild to very severe. Knowledge concerning all the parameters involved in the disease severity is still lacking. Besides the genotype of the causative hantavirus, human HLA-B8, -DR3, and -DQ2 alleles may be associated with more or less serious symptoms in HFRS (*12*). Our study included only hospitalized HFRS patients. More attention should be paid to outpatients referred to family doctors, infectious disease specialists, and nephrologists.
